# Insulin Edema Syndrome due to Rapid Glucose Correction in a Diabetic Patient

**DOI:** 10.1155/2022/3027530

**Published:** 2022-08-16

**Authors:** Siham M. Hussien, Hasan Imanli, Dena H. Tran, Robert D. Chow, Aseem Sood

**Affiliations:** Department of Medicine, University of Maryland Medical Center Midtown Campus, 827 Linden Avenue, Baltimore, MD 21201, USA

## Abstract

Edema resulting from the initiation of insulin therapy or intensification of glycemic control is a rare and under-recognized complication. In this report, we present a case of a 46-year-old patient with insulin-dependent diabetes mellitus (IDDM) who avoided insulin treatment due to associated peripheral edema. Though rare, this phenomenon is typically seen in patients with elevated glucose levels who are initiated on insulin treatment, resulting in rapid correction and tight control of glucose levels. The diagnosis of insulin-induced edema is made after other causes of acute edema are ruled out. Furthermore, in this case report, we will also discuss the postulated mechanisms for the edema-causing property of insulin.

## 1. Introduction

Insulin edema syndrome is a rare complication of insulin therapy. Edema resulting from initiation of insulin therapy or intensification of glycemic control is an under-recognized complication [1]. This phenomenon has typically been described in patients with poorly controlled diabetes mellitus during the initiation of insulin treatment, with rapid correction and tight control of glucose levels [1]. However, the rarity and lack of awareness of this phenomenon creates challenges in establishing the prevalence, making an accurate diagnosis, and creating guidelines for treatment. We report a case of insulin edema during rapid correction and strict glucose control in a diabetic patient with poorly controlled glucose levels and elevated hemoglobin A1c level, with the goal of highlighting this complication, exploring the etiology and pathophysiology of insulin-related edema, and acknowledging it as a potential reason why patients decline insulin therapy.

## 2. Case Presentation

A 46-year-old man with a medical history significant for insulin-dependent diabetes mellitus (IDDM), recurrent pancreatitis, alcohol use disorder, and hypertension presented with severe abdominal pain, nausea, and vomiting. His abdominal symptoms occurred intermittently for approximately three weeks prior to admission, during which time he had stopped using his insulin pump with continuous glucose monitoring after his device broke over two months ago. Ten days before his current admission, he was admitted to the hospital for diabetic ketoacidosis during which time he was transitioned to subcutaneous insulin and discharged on insulin detemir 8 units twice daily, insulin lispro 8 units 3 times daily with meals, calcium carbonate 750 mg every 6 hours, pancrelipase 12,000-unit capsule 3 times daily, sucralfate 10 ml four times daily, and trazodone 50 mg tablet nightly. He reported stopping the use of his insulin because he believed it was causing bilateral pedal edema to the level of his knees. He noted consistent patterns of developing edema in his lower extremities a few days after insulin administration. The patient stated that the swelling in his legs resolved after cessation of insulin use. During his hospital admission, the patient refused diabetes education and insisted on the side effect of insulin as a reason for non-compliance with treatment routine.

Social history was remarkable for 60 pack-year tobacco use, daily marijuana use, and no other illicit drug use. Review of systems was remarkable for recent weight loss due to poor diet, chronic smoker's cough, abdominal pain, nausea, vomiting, polyuria, polydipsia, and lower extremity bilateral pedal edema present on days of insulin use that resolves when the patient does not use his insulin. He also reported having chronic diabetic neuropathy and numbness in his feet bilaterally.

**Figure 1:**
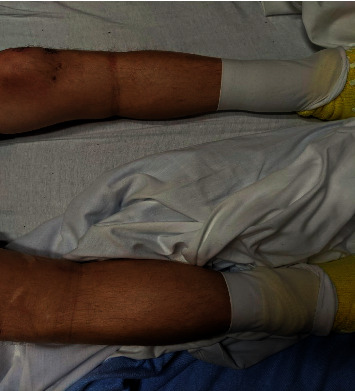
Bilateral pedal edema to the level of the knees secondary to insulin therapy (photograph was taken with patient's permission).

Physical examination was notable for temperature of 98.2 degrees Fahrenheit, blood pressure of 151/76, heart rate of 88, respiratory rate of 20, and oxygen saturation of 98 percent on room air. Cardiovascular examination revealed normal rate and regular rhythm, there was no elevated jugular venous pressure, and lung sounds were with no rales. He had generalized abdominal tenderness on palpation without guarding, rebound tenderness, or distension. There was bilateral pedal pitting 4+ edema up to his knees (Figure 1).

Laboratory results were consistent with diabetic ketoacidosis (DKA): anion gap of 23, bicarbonate of 11 mmol/L, sodium of 130 mmol/L, chloride of 93 mmol/L, creatinine of 0.41 mg/dL, glucose of 374 mg/dL, total protein of 5.4 g/dL, albumin of 3.4 g/dL, and positive beta hydroxybutyrate of 2.74, and urinalysis showed 1+ ketones and 3+ glucose and was negative for proteinuria. Computed tomography of the abdomen and pelvis was remarkable for scattered calcification along the atrophic pancreas suggesting chronic pancreatitis and normal liver texture. Lower extremity venous Doppler was not done due to lack of recent immobility, negative Homans' sign, bilaterality, and low clinical suspicion for deep venous thrombosis. Transthoracic echo was unremarkable without any ventricular systolic or diastolic dysfunction.

## 3. Discussion

Insulin edema syndrome is an adverse effect of insulin therapy ensuing from the rigorous correction of hyperglycemia in patients with poorly controlled diabetes mellitus [2]. In addition to poor glycemic control, having a low body mass index increases the risk of developing insulin edema [3]. It is also seen in younger patients with newly diagnosed type 1 diabetes mellitus. The edema is generally mild, affecting the lower extremities. However, there have been reports of severe presentations causing anasarca, ascites, and pleural effusion [2].

The cause of our patient's insulin-dependent diabetes was believed to be secondary to his history of recurrent pancreatitis in the setting of alcohol abuse, also known as pancreatogenic diabetes or Type 3c [4]. Insulin edema may have variable presentations, from benign to frank heart or renal failure [2, 5].

The edema has been postulated to result from several mechanisms, including increasing capillary permeability and renal salt retention, thus inducing extravasation of fluid, resulting in edema [6]. Insulin is known to cause the reabsorption of sodium in the kidneys by stimulating the Na^+^/K+-ATPase as well as expression of Na^+^/H^+^ exchanger 3 in the proximal tubule, and it also causes vasodilation, which contributes to fluid retention. Hyperglycemia independently also causes an increase in vascular permeability, which is consistent with the development of edema in patients that have persistently elevated glucose levels [3]. Persistently elevated glucose levels in diabetic patients cause poor integrity of the vascular membrane, which increases the risk of edema as a result of rapid serum osmolar changes. This proposed mechanism is supported by the phenomenon of “refeeding edema.” Refeeding edema occurs with an increase in endogenous plasma insulin associated with carbohydrate ingestion after prolonged starvation [7].

There are different approaches to treating insulin edema, ranging from conservative management with restriction of salt and liquid intake to pharmacotherapy with diuretics, epinephrine, or ephedrine in refractory cases [8]. Adjusting the dose of insulin has also been shown to reduce the degree of edema [8]. As discussed above, the patient in this presentation noticed improvement of the leg swelling with cessation of insulin usage, which unfortunately resulted in his non-compliance and several sequelae of hospital admission as a result of DKA. During his admission, he received 20 mg of furosemide daily with rapid improvement in his symptoms.

## 4. Conclusion

Insulin edema syndrome is a rare complication of insulin therapy. Appropriate patients who present with edema should have this clinical entity as an element in their differential diagnoses. Potential complications involving the cardiovascular and pulmonary systems should be considered. After other cardiac, hepatic, vascular, and nephrotic causes of sudden onset edema have been excluded, it is reasonable to attribute the etiology to this under-recognized phenomenon. Awareness of this condition can give patients reassurance and confidence in their insulin regimen.

## Data Availability

No data were used to support this study.
